# Development of a High-Performance *Trichoderma* Mutant for Enhanced Cellulase Production Through UV-Induced Random Mutagenesis

**DOI:** 10.3390/jof11060439

**Published:** 2025-06-09

**Authors:** Seungjun Kim, Iksu Ha, Yun-Yeong Lee, Junseo Lee, Jeonghee Yun

**Affiliations:** Department of Forest Products and Biotechnology, Kookmin University, Seoul 02707, Republic of Korea; sj99ksj@kookmin.ac.kr (S.K.); gk257252@kookmin.ac.kr (I.H.); yunlee529@kookmin.ac.kr (Y.-Y.L.); lmn9022@kookmin.ac.kr (J.L.)

**Keywords:** UV-induced mutagenesis, *Trichoderma* sp., cellulase production, lignocellulosic biomass, bioreactor

## Abstract

Ultraviolet (UV)-induced mutagenesis is a cost-effective and straightforward technique for introducing random genetic variations without the use of chemical reagents or genetic engineering. It is commonly employed to enhance enzyme activity in industrial trains. In this study, *Trichoderma* sp. was exposed to UV radiation at varying distances (4, 9, and 13 cm) and durations (2, 4, 6, and 8 min) to induce mutations. The activities of endoglucanase (EG), β-glucosidase (BGL), and cellobiohydrolase (CBH) were assessed following treatment. The 4 cm exposure distance yielded the highest enhancement, with EG, BGL, and CBH activities increasing 1.5-, 1.3-, and 0.9-fold, respectively. When the distance was fixed at 4 cm, the optimal exposure time was identified as 4 min, yielding further enhancements of 1.9-, 1.6-, and 1.4-fold, respectively. The resulting mutant, designated Mut-4, was scaled up in a 10-L bioreactor to assess its industrial applicability. Mut-4 retained its enhanced performance, achieving 1.9-, 2.0-, and 1.4-fold enhancements in EG, BGL, and CBH activities, respectively, compared with the original strain. These findings indicate that combining UV-induced mutagenesis with basic screening is an effective strategy for enhancing cellulolytic enzyme production, representing a promising approach for lignocellulosic biomass conversion.

## 1. Introduction

Cellulases are essential enzymes involved in the conversion of lignocellulosic biomass into monosaccharides, which can subsequently be processed into biofuels and other high-value products [1,2]. This enzyme complex facilitates the breakdown of the highly recalcitrant structure of lignocellulosic materials—which are abundant and renewable resources [3,4,5]. The growing demand for sustainable energy sources has intensified research aimed at optimizing cellulase production, particularly through microbial systems [6,7,8].

Among the various microorganisms employed for cellulase production, *Trichoderma* species are particularly well known [9]. These fungi are recognized for their high efficiency in enzyme synthesis, making them valuable for industrial and biotechnological applications. *Trichoderma* sp. produces a synergistic suite of cellulases, a group of enzymes that depolymerize cellulose into glucose. This includes the primary hydrolytic enzymes endoglucanase (EG), cellobiohydrolase (CBH), and β-glucosidase (BGL), as well as accessory proteins such as lytic polysaccharide monooxygenases and cellobiose dehydrogenase, which contribute to cellulose degradation through oxidative mechanisms and electron transfer [10]. Among these, EG, CBH, and BGL play crucial roles in the hydrolytic cleavage of cellulose and are commonly used as key performance indicators in strain improvement studies. Specifically, EG catalyzes the random cleavage of internal β-1,4-glycosidic bonds in cellulose chains, generating new chain ends. CBH acts on these chain ends to release cellobiose units, and BGL subsequently hydrolyzes cellobiose into glucose [11,12]. This synergistic action is crucial for the efficient saccharification of lignocellulosic biomass.

However, despite the potential of these enzymes, their high production cost remains a significant barrier, substantially increasing overall processing expenses and reducing their economic feasibility for large-scale applications [13,14]. These limitations underscore the need for continued research to enhance cellulase productivity and activity.

To address these challenges, the genetic modification of microorganisms has gained attention as a promising strategy [15,16]. In particular, ultraviolet (UV)-induced mutation has emerged as a relatively simple yet effective method for generating microbial mutants with enhanced enzyme activity [17]. UV radiation acts as a potent mutagen by inducing DNA damage, which can lead to genetic mutations [18]. These mutations may improve physiological traits, such as increased enzyme production—an essential goal in microbial strain improvement [16].

Biochar, a carbon-rich material derived from the thermal degradation of biomass, has been shown to support microbial growth and metabolic activity, potentially enhancing enzyme production [19,20,21]. A previous study conducted by this research group reported a synergistic effect between *Trichoderma* sp. and biochar, resulting in a significant increase in cellulase production [22]. This enhancement was attributed to the increased availability of substrate and the stimulation of microbial activity facilitated by biochar [23].

Building on these findings, our study aimed to enhance cellulase production by applying UV-induced random mutagenesis to *Trichoderma* sp. KMF006. Mutations were induced by systematically varying the distance between the UV light source and the fungal culture (4, 9, and 13 cm) and adjusting the exposure time (2, 4, 6, and 8 min). The resulting mutants were screened for cellulase activity and stability, and a single strain exhibiting superior enzymatic performance was selected. This top-performing mutant was further evaluated through a fermentation scale-up in a 10-L bioreactor, where cellulase activity and protein concentration were assessed under optimized conditions. Biochar supplementation was also incorporated as a supportive factor to enhance fungal performance. Collectively, this study examines the combined application of UV-induced mutagenesis and biochar-assisted fermentation as a strategy for enhancing cellulase production by *Trichoderma* sp., with the ultimate goal of enhancing enzymatic saccharification efficiency for lignocellulosic biomass conversion.

## 2. Materials and Methods

### 2.1. Microorganisms and UV Mutagenesis

The patented *Trichoderma longibrachiatum* strain KMF006 (KCTC13500BP) [24], a well-established cellulase-producing fungus, was selected for UV-induced mutagenesis in this study. The strain was cultured on malt extract agar (MEA) and incubated at 30 °C for 7 days. Fully grown cultures were stored at 4 °C until further use.

**Figure 1:**
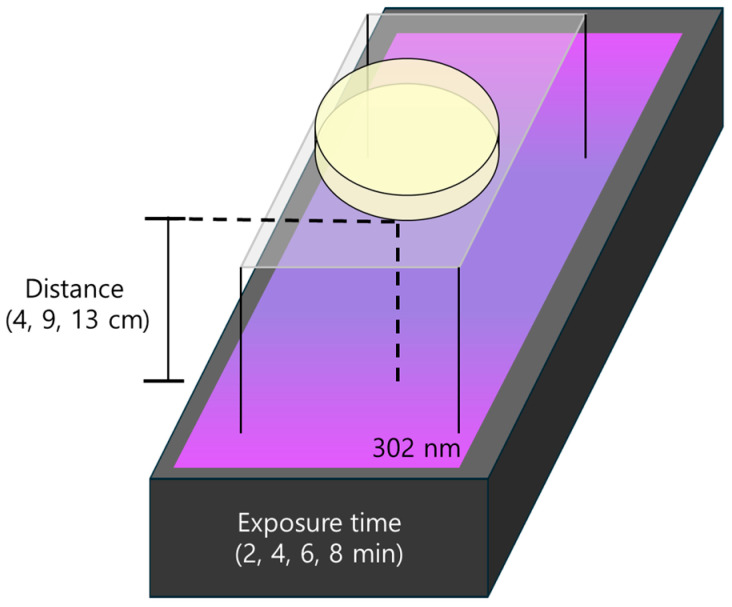
Experimental setup for UV-induced mutagenesis.

UV-induced mutagenesis was performed using mature fungal cultures grown on MEA plates. The plates were uncovered and exposed to UV light emitted by a transilluminator (TI2621BS, Avegene Life Science, Taipei, Taiwan) at a wavelength of 302 nm. Exposure was conducted in a dark room to prevent interference from ambient light. Mutagenesis conditions were systematically varied by adjusting the distance between the UV source and the culture surface (4, 9, and 13 cm) and the exposure duration (2, 4, 6, and 8 min). A schematic diagram of the experimental setup is presented in Figure 1.

### 2.2. Cultivation of Mutant Strains for Activity Screening

Flask cultivation was conducted to evaluate the cellulase-producing capacity of the UV-mutated strains under submerged fermentation conditions. Biochar supplementation was included based on previous studies reporting its positive effects on cellulase activity enhancement [22]. The preculture was prepared in 250 mL baffled flasks containing 100 mL of potato dextrose broth. After UV-induced mutagenesis, colonies grown on MEA plates were used to excise a single fungal pellet (1 cm × 1 cm) from a region equidistant from the colony center. The pellet was transferred to the center of a fresh MEA plate for subculturing. Following subculture, 10 circular fungal pellets (1 cm × 1 cm) were excised from the same radial distance using sterile pipette tips and inoculated into flasks containing liquid medium. The cultures were incubated at 27 °C and 150 rpm for 5 days.

The primary culture medium was prepared based on a formulation by Choi (unpublished), consisting of 15 g⋅L^−1^ yeast extract, 5 g⋅L^−1^ KH_2_PO_4_, 5 g⋅L^−1^ K_2_HPO_4_, 3 g⋅L^−1^ MgSO_4_ 7H_2_O, and 3% (*w*/*v*) Avicel. The initial pH was adjusted to 5.0. Biochar was added at a concentration of 2% (*w*/*v*), consistent with the optimal level reported in previous studies [20]. Each flask was sterilized at 121 °C for 30 min and inoculated with 5% (*v*/*v*) preculture. The cultures were incubated at 31.3 °C and 180 rpm for 21 days to evaluate enzyme activity and protein concentration.

### 2.3. Enzyme Extraction and Activity Assays

Enzyme activity and protein concentration were evaluated during flask cultivation, with sampling conducted every 3 days. For sample preparation, 1 mL of the culture broth was transferred to a 1.5 mL sterile microcentrifuge tube and centrifuged at 13,000 rpm for 10 min at 4 °C. The supernatant was collected and either used immediately or stored at 4 °C for subsequent enzyme and protein assays.

#### 2.3.1. Endoglucanase

EG activity was measured using the Somogyi–Nelson method, with 2% (*w*/*v*) carboxymethylcellulose (CMC) in 0.1 M sodium citrate buffer (pH 5.0) as the substrate [25]. A reaction mixture containing 45 μL of CMC and 5 μL of the enzyme solution was incubated at 60 °C for 30 min. To terminate the reaction, 50 μL of copper reagent was added, and the mixture was heated in boiling water for 10 min. Subsequently, 50 μL of the Nelson reagent and 850 μL of distilled water were added. The absorbance was measured at 650 nm using a UV/Vis spectrophotometer (Synergy LX multi-mode reader, BioTek, Agilent Technologies, Winooski, VT, USA). One unit (U) of enzymatic activity was defined as the amount of enzyme required to release 1 μmol of glucose per minute under the assay conditions. All experiments were performed in duplicate.

#### 2.3.2. β-Glucosidase

BGL activity was determined using 10 mM p-nitrophenyl-β-D-glucopyranoside in 0.1 M sodium citrate buffer (pH 5.0). The 200-μL reaction mixture consisted of 160 μL of buffer, 20 μL of substrate, and 20 μL of enzyme. After incubation at 65 °C for 15 min, the reaction was stopped by adding 50 μL of 1 M Na_2_CO_3_. Absorbance was measured at 405 nm using a spectrometer [26]. One unit (U) of enzyme activity was defined as the amount of enzyme required to produce 1 μmol of p-nitrophenol per minute. All experiments were performed in duplicate.

#### 2.3.3. Cellobiohydrolase

CBH activity was measured using 10 mM p-nitrophenyl-β-D-cellobioside under the same conditions as the BGL assay [26]. The reaction was terminated by adding 50 μL of 1 M Na_2_CO_3_, and the absorbance was measured at 405 nm. One unit (U) of enzyme activity was defined as the amount of enzyme required to produce 1 μmol of p-nitrophenol per minute. All experiments were performed in duplicate.

#### 2.3.4. Protein Concentration

Protein concentration was determined using a Bradford-based protein assay kit (Bio-Rad, Hercules, CA, USA). The reaction mixture consisted of 400 μL of distilled water, 100 μL of protein assay reagent, and 10 μL of the enzyme solution. After incubation at room temperature for 5 min, absorbance was measured at 595 nm. Protein concentrations were determined using a standard curve generated with bovine serum albumin (BSA). All experiments were performed in duplicate.

### 2.4. Identification of the Optimally UV-Mutated Strain

Based on the enzyme activity screening results, the mutant strain exposed to UV light at a distance of 4 cm for 4 min exhibited the highest cellulase activity among all tested conditions. This strain was selected for further characterization and designated as Mut-4.

To determine its taxonomic position, internal transcribed spacer (ITS) region sequencing was performed by Macrogen (Seoul, Republic of Korea). The ITS region, located between the conserved 18S and 28S rRNA genes, was amplified using the ITS5 (5′-TCC GTA GGT GAA CCT GCG G-3′) and ITS4 (5′-TCC TCC GCT TAT TGA TAT GC-3′) primers. The amplified product (638 bp) was sequenced, and the resulting sequence was compared with the National Center for Biotechnology Information (NCBI database).

**Figure 2:**
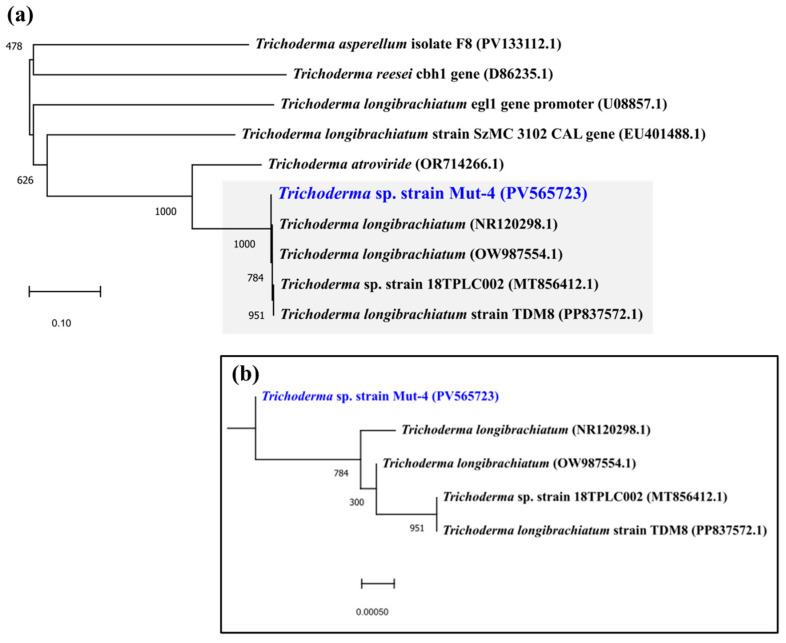
Maximum likelihood phylogenetic tree of *Trichoderma* sp. based on ITS region sequences. (**a**) Full tree showing the phylogenetic relationships among the analyzed strains. The scale bar represents 0.10 nucleotide substitutions per site. (**b**) Enlarged view of the clade containing the *Trichoderma* sp. strain Mut-4 (accession no PV565723) alongside closely related *T. longibrachiatum* strains.

The ITS sequence of Mut-4 showed 99.84% identity with *Trichoderma longibrachiatum* in the NCBI database. Phylogenetic analysis based on ITS sequences further confirmed this identification, placing Mut-4 within the *T. longibrachiatum* clade (Figure 2). The sequence has been deposited in the NCBI GenBank database under accession number PV565723.

**Figure A1:**
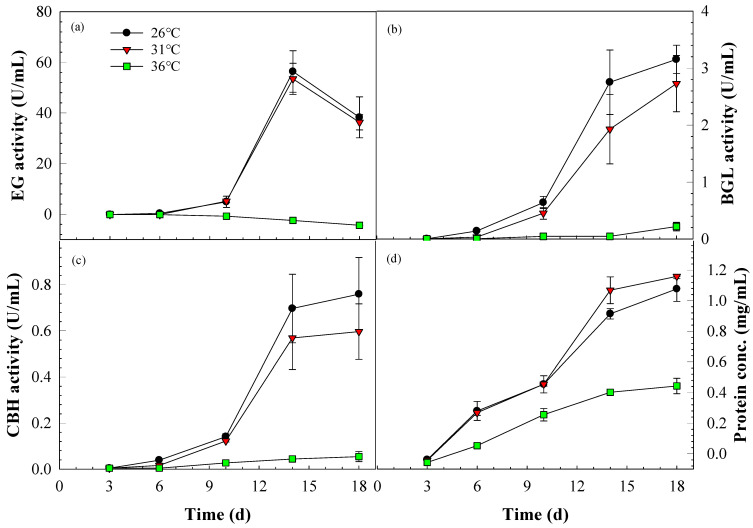
Temperature-dependent data showing (**a**) endoglucanase (EG) activity, (**b**) β-glucosidase (BGL) activity, (**c**) cellobiohydrolase (CBH) activity, and (**d**) protein concentration of *Trichoderma* sp. Mut-4 cultivated at 26 °C, 31 °C, and 36 °C.

As previously reported by Kredics et al., *T. longibrachiatum* may exhibit opportunistic pathogenicity under certain conditions [27]. To address this potential risk, we evaluated the thermal sensitivity of the mutant strain. Cultivation was conducted at elevated temperatures (above 35 °C) to examine the viability of the strain under conditions approximating human body temperature. The mutant showed a marked decline in both growth and enzymatic activity above 35 °C, suggesting a low likelihood of survival or pathogenic behavior under physiological conditions. The corresponding data are presented in Appendix A Figure A1.

### 2.5. 10-L Bioreactor for Cellulase Production

To evaluate cellulase production under bioreactor conditions, *Trichoderma* sp. Mut-4—isolated under optimal UV treatment (4 cm distance; 4 min exposure)—was cultivated in a 10 L stirred-tank bioreactor (working volume: 6 L) under submerged fermentation.

The culture medium comprised 10 g⋅L^−1^ yeast extract, 5 g⋅L^−1^ KH_2_PO_4_, 5 g⋅L^−1^ K_2_HPO_4_, and 3 g⋅L^−1^ MgSO_4_·7H_2_O, supplemented with 1.5% (*w*/*v*) Avicel and 0.5% (*w*/*v*) cellulose as carbon sources. The initial pH was adjusted to 5.0 before sterilization. Based on flask-scale optimization, 2% (*w*/*v*) biochar was also added. The bioreactor was inoculated with 5% (*v*/*v*) of a *Trichoderma* sp. Mut-4 seed culture was operated at 31.3 °C for 15 days, with aeration maintained at 2 L·min^−1^.

### 2.6. Statistical Analysis

For each experimental condition, two independent cultures (biological replicates) were prepared. Enzyme activity for each culture was measured in triplicate (technical replicates) using repeated absorbance readings. Thus, data for each condition were expressed as the mean ± standard deviation (SD), with a total of *n* = 6 measurements per condition.

Statistical analyses were conducted using R software (Version 4.4.1). A multifactorial analysis was conducted to identify significant differences, with a *p*-value of <0.05 considered statistically significant. Prior to the analysis, assumptions of normality and homogeneity of variance were assessed. When both assumptions were satisfied, one-way ANOVA was applied, followed by Tukey’s HSD post-hoc test.

Enzyme activity data from the 10-L bioreactor experiment were further analyzed using SigmaPlot software (SigmaPlot software (Version 12.5, Systat Software, Inc., San Jose, CA, USA) to model the temporal production profile. A three-parameter Gaussian nonlinear regression model was fitted to the data, as described by Equation (1).

(1) $$y=a\;\cdot \;e^{-\frac{{\left(x-b\right)}^{2}}{{2c}^{2}}}$$

Here *y* is the enzyme activity, *x* is the incubation time, *a* represents the maximum activity, *b* is the peak center (i.e., time of maximum activity), and *c* is the peak width. The constant *e* denotes Euler’s number (approximately 2.718), which is the base of the natural logarithm and is used in the exponential component of the model. This model was used to estimate the peak activity time point and the distribution pattern of EG, CBH, and BGL over the 15-day cultivation period in the bioreactor.

## 3. Results

### 3.1. Effect of UV Light Exposure Distance on Cellulase Activity

**Figure 3:**
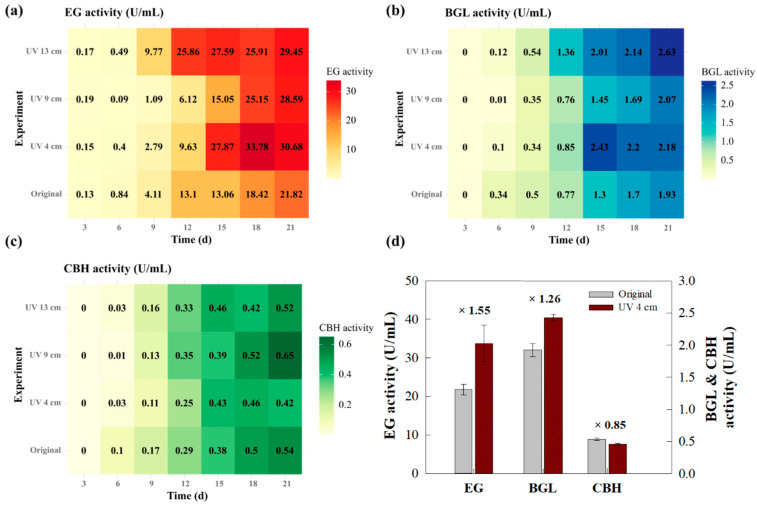
Comparative analysis of cellulase activities under different UV exposure distances. Heatmaps showing the enzyme activities of (**a**) EG, (**b**) BGL activity, and (**c**) CBH activity over the cultivation period (3–21 days). Each cell represents the measured activity (U·mL^−1^) at a given time point, with bold values indicating the highest activity observed under each treatment. (**d**) Maximum enzyme activities of the 4-cm UV-exposed strain compared with the original strain. X-fold values indicate a relative improvement in the maximum activity of the UV 4 cm-exposed strain compared with the original strain. Error bars represent the standard deviation (SD) of three independent replicates (*n* = 3). Different letters denote statistically significant differences (*p* < 0.05) based on one-way ANOVA followed by Tukey’s HSD test.

**Figure A2:**
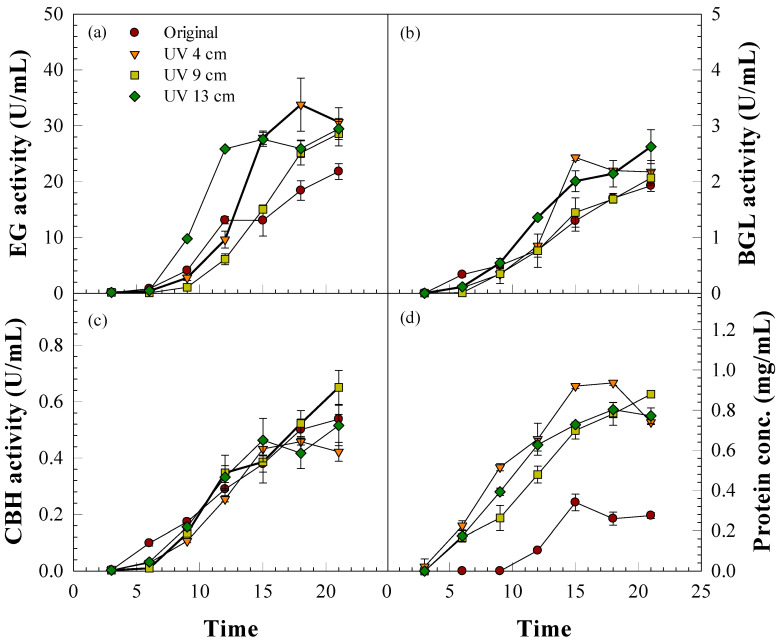
Effective UV exposure distance on (**a**) EG activity, (**b**) β-BGL activity, (**c**) CBH activity, and (**d**) protein concentration for the original and UV-mutated strains.

The effect of the UV light exposure distance on enzyme activity was investigated over a 21-day cultivation period. Appendix A Figure A2 presents the time-course profiles of the enzyme activities, showing that the strain exposed to UV at a distance of 4 cm exhibited faster onset and higher levels of enzyme expression, particularly during the exponential growth phase. These results suggest that UV exposure at 4 cm more effectively induced enzyme production compared with other distances. Furthermore, the strain exposed to UV at 4 cm distance showed the highest protein concentration, reaching 0.936 mg·mL^−1^ on day 15, indicating enhanced overall productivity. The maximum enzyme activity values and their corresponding time points are summarized in Figure 3 and Appendix A Table A1.

Heatmap analysis (Figure 3a–c) was used to visualize the activity trends of EG, BGL, and CBH across different UV exposure distances. Color intensity reflects enzyme activity levels, with darker shades indicating higher activity. For EG, the strain exposed to UV at 4 cm exhibited the highest activity (33.784 U·mL^−1^ on day 18), followed by the strains exposed at 13 and 9 cm. Conversely, the original strain peaked at a lower activity level (21.824 U·mL^−1^). BGL activity also reached its maximum in the UV 4 cm-exposed strain (2.432 U·mL^−1^ on day 15), exceeding that of the original strain (1.926 U·mL^−1^). CBH activity showed a different trend, with the highest value observed in the UV 9 cm-exposed strain (0.651 U·mL^−1^ on day 18). Nevertheless, the UV 4 cm-exposed strain demonstrated higher CBH activity than the original strain, with values of 0.458 and 0.379 U·mL^−1^, respectively, on day 15. As shown in Figure 3d, the UV 4 cm-exposed strain exhibited 1.55-fold and 1.26-fold increases in EG and BGL activities, respectively, compared with the original strain. The CBH activity was 0.85 times that of the original strain. These differences were statistically significant (*p* < 0.05), confirming that UV exposure at a distance of 4 cm was the most effective in enhancing enzyme activity.

**Figure 4:**
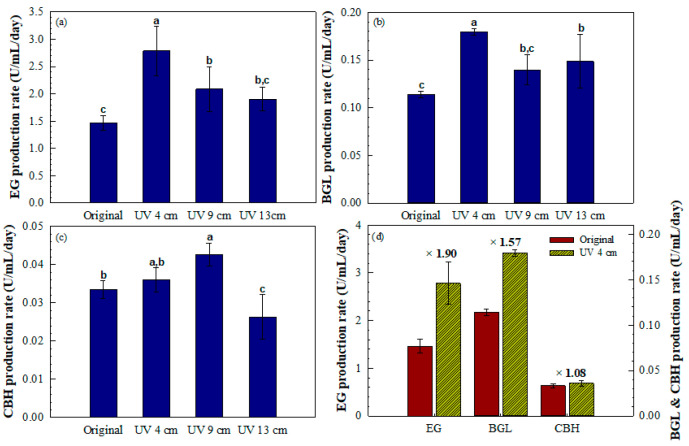
Comparative analysis of cellulase production rates under different UV exposure distances. Bar graphs showing the enzyme production rates of (**a**) EG, (**b**) BGL, and (**c**) CBH, measured between 6 and 18 days post-inoculation, corresponding to the exponential growth phase. (**d**) Relative increase in enzyme production rates of the strain exposed to UV at 4 cm compared with the original strain. X-fold values indicate the enhancement achieved through UV mutagenesis. Error bars represent the SD of three independent replicates (*n* = 3). Different letters indicate statistically significant differences (*p* < 0.05) based on one-way ANOVA followed by Tukey’s HSD test.

The enzyme production rates of EG, BGL, and CBH during the exponential growth phase (6–18 days post-inoculation) are presented in Figure 4a–c. The highest EG production rate was observed in the strain exposed to UV at 4 cm (approximately 3.2 U·mL^−1^·day^−1^), followed by the UV-exposed strains at 9 and 13 cm, and the original strain (Figure 4a). A similar trend was observed for BGL, with the UV 4 cm-exposed strain exhibiting the highest production rate (approximately 0.18 U·mL^−1^·day^−1^) (Figure 4b). For CBH, the highest production rate was recorded in the UV 9 cm-exposed strain, whereas the UV 4 cm-exposed strain still showed a higher rate than the original strain. The UV 13 cm-exposed strain exhibited the lowest CBH production rate among all treatments (Figure 4c). Figure 4d illustrates the relative fold increase in enzyme production rates compared with the original strain. The UV 4 cm-exposed strain exhibited a 1.90-, 1.57-, and 1.08-fold increase in EG, BGL, and CBH production rates, respectively. Among the tested UV exposure conditions, the 4 cm distance resulted in the highest enhancement of EG and BGL production during the exponential growth phase.

### 3.2. Effect of UV Exposure Time on Cellulase Activity and Protein Concentration

**Figure A3:**
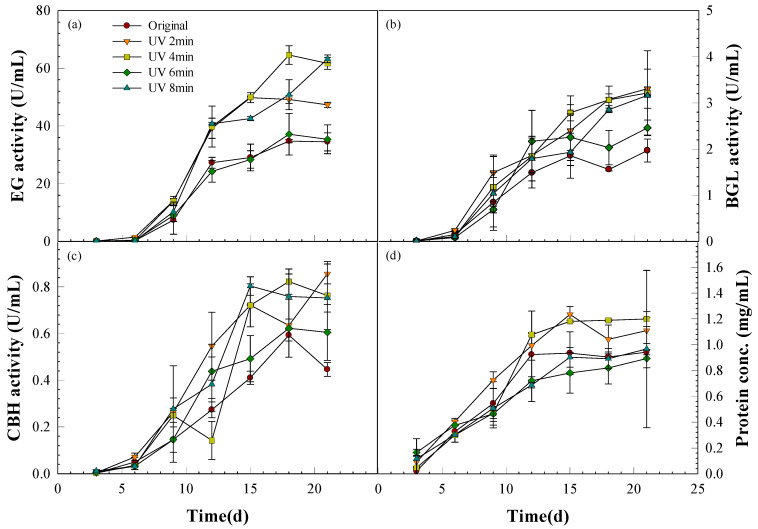
Time-course profiles of (**a**) endoglucanase (EG) activity, (**b**) BGL activity, (**c**) CBH activity, and (**d**) protein concentration for the original and UV-mutated strains.

In the previous experiment, UV exposure at a distance of 4 cm resulted in the highest overall activity. Based on this finding, the exposure distance was fixed at 4 cm, and the effects of different exposure durations (2, 4, 6, and 8 min) on cellulase activity and protein concentration were subsequently evaluated. The results are shown in Appendix A Figure A3 and Table 1. For EG activity, the 4 min exhibited the highest value (64.599 U·mL^−1^ on day 18), followed closely by the 8 min exposure (63.251 U·mL^−1^ on day 21). The 2 min exposure resulted in an activity of 49.789 U·mL^−1^ on day 15. In comparison, the original strain reached a maximum EG activity of 34.750 U·mL^−1^ on day 18. BGL activity was highest in the 2-min exposed strain (3.309 U·mL^−1^), followed by the 4-min (3.217 U·mL^−1^) and 8-min exposure strains (3.165 U·mL^−1^). The original strain recorded a BGL activity of 1.970 U·mL^−1^. For CBH activity, the 2-min exposed strain also showed the highest value (0.856 U·mL^−1^), followed by the 4-min (0.823 U·mL^−1^) and 8-min (0.804 U·mL^−1^) exposures. The original strain exhibited a maximum CBH activity of 0.593 U·mL^−1^. The protein concentration was also highest in the 2-min-exposed strain (1.234 mg·mL^−1^ on day 15). The 4-min- and 8-min-exposed strains showed values of 1.999 and 0.966 mg·mL^−1^, respectively. The original strain recorded a concentration of 0.942 mg·mL^−1^. The maximum enzyme activity values and corresponding sampling days are listed in Table 1. Statistically significant differences (*p* < 0.05) were observed between the original and UV-treated strains.

**Figure 5:**
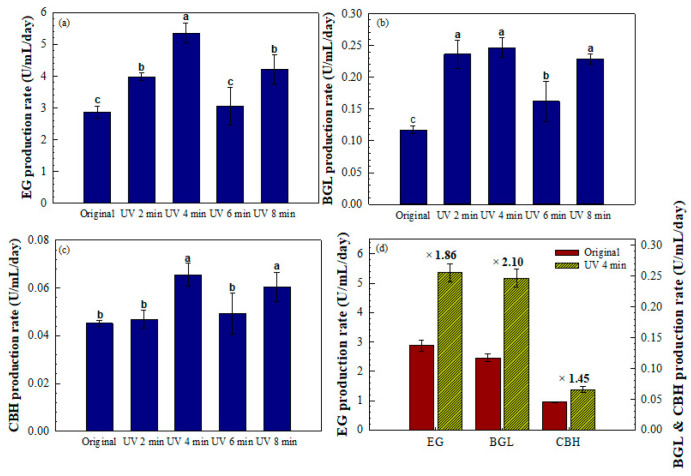
Comparative analysis of cellulase production rates under different UV exposure durations. Bar graphs showing the enzyme production rates of (**a**) EG, (**b**) BGL, and (**c**) CBH, measured during the exponential growth phase (6–18 days) following UV mutagenesis at varying exposure times. (**d**) Relative increase in the enzyme production rates of the strain exposed to UV for 4 min compared with the original strain. X-fold values show the enhancement achieved through UV mutagenesis. Error bars indicate the SD of three independent replicates (*n* = 3). Different letters denote statistically significant differences (*p* < 0.05) based on one-way ANOVA followed by Tukey’s HSD test.

In addition, Figure 5 illustrates the cellulase production rates during the exponential growth phase (6–18 days). The strain exposed to UV for 4 min exhibited the highest production rates for all three enzymes: EG at 5.4 U·mL^−1^·day^−1^, BGL at 0.26 U·mL^−1^·day^−1^, and CBH at 0.065 U·mL^−1^·day^−1^. As shown in Figure 5d, these correspond to 1.86-, 2.10-, and 1.45-fold increases in EG, BGL, and CBH, respectively, relative to the original strain. These results indicate that the 4 min UV-exposed strain achieved the most rapid and consistent enzyme production across all evaluated components. Therefore, UV exposure at 4 cm for 4 min was selected as the optimal mutagenesis condition. The mutant obtained under this condition was designated as strain Mut-4 for subsequent analysis. Furthermore, the enhanced enzyme activities of the Mut-4 strain were stably maintained over at least 10 successive generations, indicating the genetic stability of the UV-induced mutation.

### 3.3. Enzyme Activity and Protein Concentration in a 10-L Bioreactor

**Figure 6:**
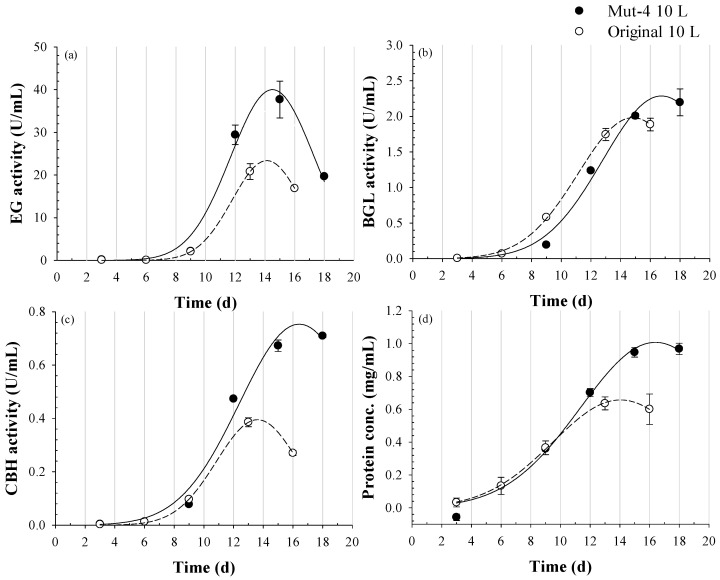
Regression analysis predicting maximum cellulase activities and protein concentration in a 10 L bioreactor for the original and *Trichoderma* sp. Mut-4 strains. Time-course data of enzyme activities and protein production were fitted using Gaussian regression to evaluate fermentation performance. (**a**) endoglucanase (EG) activity, (**b**) β-glucosidase (BGL) activity, (**c**) cellobiohydrolase (CBH) activity, and (**d**) protein concentration.

The Mut-4 strain was cultivated in a 10 L stirred-tank bioreactor to evaluate its enzyme production under pilot-scale conditions. Enzyme activities for EG, BGL, and CBH, along with protein concentration, were monitored throughout the cultivation period. A Gaussian three-parameter regression model was used to describe the production kinetics (Figure 6). All models demonstrated an excellent fit, with R^2^ > 0.99 and *p* < 0.05, indicating high model reliability. For the Mut-4 strain, the maximum EG activity reached 40.01 U·mL^−1^ at 14.50 days. BGL activity peaked at 2.29 U·mL^−1^ on day 16.75, and CBH reached 0.75 U·mL^−1^ on day 16.42 (Figure 6a–c). Protein concentration also reached its peak at 1.01 mg·mL^−1^ on day 16.40 (Figure 6d). In contrast, the original strain showed lower enzyme activities and earlier peak times: EG peaked at 23.38 U·mL^−1^ at 14.12 days, BGL at 1.98 U·mL^−1^ at 14.83 days, and CBH at 0.40 U·mL^−1^ at 13.60 days (Figure 6a–c). The maximum protein concentration in the original strain was 0.66 mg·mL^−1^ at 14.03 days (Figure 6d).

Compared with the original strain, Mut-4 exhibited significantly higher enzyme activities and protein concentration in the 10 L bioreactor, with maximum values showing 1.7-, 1.15-, 1.91-, and 1.53-fold increases for EG, BGL, CBH, and protein concentration, respectively. Although the peak values for enzyme activities and protein concentration were slightly later than those of the original strain, Mut-4 consistently maintained higher levels throughout the cultivation period. Notably, when both strains were compared at day 14—the peak time point for the original strain—Mut-4 had already surpassed the maximum values of the original strain. This indicates that the improvements in enzyme production were not only quantitative but also applicable within shorter cultivation periods. These results highlight the enhanced performance and potential industrial scalability of Mut-4 for enzyme production.

## 4. Discussion

UV mutagenesis was selected in this study owing to its operational simplicity, short treatment duration, and lower safety concerns compared with chemical mutagens, such as ethyl methane sulfonate (EMS) and methyl methane sulfonate (MMS). Although EMS and MMS are commonly used to induce point mutations, their application involves hazardous handling, additional neutralization procedures, and higher toxicity concerns [16]. Conversely, UV treatment does not require hazardous reagents and is easily applicable for large-scale mutant screening, making it more suitable for initial microbial strain improvement and bioreactor-scale studies.

Given these advantages, this study focused on optimizing UV mutagenesis conditions by adjusting exposure distance and duration to enhance cellulase activity. The findings revealed enzyme-specific sensitivities and nonlinear activity trends in response to varying exposure times, highlighting the need for precisely calibrated UV protocols in industrial strain development. Different cellulase components exhibited distinct responses to UV exposure distances. EG and BGL showed peak activity at 4 cm, while CBH activity was highest at 9 cm. These differences are likely attributed to the structural and regulatory variations among the enzymes. According to Shahbazi et al. [18], the cellulase system of *Trichoderma reesei* comprises 60–80% CBH, 20–36% EG, and <1% BGL. CBHs are encoded by complex genes, such as cel7A and cel6A, making them more susceptible to damage from intense UV exposure. Conversely, EG and BGL possess simpler gene architectures and more robust transcriptional systems, which may enable them to tolerate intense UV-induced stress more effectively. This trend is consistent with the observations of Pérez et al. [28], who reviewed the structural composition and biodegradation mechanisms of lignocellulosic biomass, and Rana [29], who examined the substrate specificity of cellulase components during enzymatic hydrolysis. Both studies reported that CBHs act primarily on crystalline cellulose and exhibit structural sensitivity, whereas EGs target amorphous regions and display greater flexibility.

In recent years, non-hydrolytic accessory proteins, particularly expansin-like molecules known as swollenins, have attracted growing interest due to their ability to disrupt hydrogen bonding within cellulose microfibrils and thereby increase substrate accessibility. When used alongside cellulases, these proteins have been shown to substantially enhance hydrolytic efficiency. For example, TlSWO from *Talaromyces leycettanus* and ThSWO from *Trichoderma harzianum* significantly increased reducing sugar yields in enzymatic reactions [30,31]. Yao et al. also reported enhanced saccharification in cellulolytic fungi through the overexpression of swollenin [32], while Qin et al. recommended incorporating such proteins into engineered enzyme mixtures to improve biomass deconstruction at an industrial scale [33].

Taken together, these findings suggest that swollenin-like proteins may function as key modulators of cellulase performance, especially under optimized reaction conditions. The substantial improvement in saccharification observed in our UV-mutated strain raises the possibility that accessory factors of this nature played a contributing role. Although we were unable to directly confirm this hypothesis within the scope of the present study, it offers a promising direction for future investigation into the molecular basis of the observed enhancement. Future studies should prioritize the analysis of gene expression patterns and biological roles of swollenins in mutant strains to clarify their potential synergistic interactions with core cellulases.

Such inquiry is well aligned with our overarching research objective, advancing saccharification efficiency through UV-induced mutagenesis, and reinforces the importance of expanding future enzyme system designs to include non-hydrolytic proteins as strategic enhancers of biomass conversion.

Additionally, a nonlinear trend in cellulase activity was observed across varying UV exposure durations, with activity peaking at 4 min, declining at 6 min, and rebounding at 8 min. This pattern is likely owing to the balance between mutation accumulation and cellular viability. Rana [28] noted that prolonged UV exposure can lead to deleterious mutations that impair enzyme productivity, which may explain the decreased activity at 6 min. However, the resurgence at 8 min could reflect the selective survival of rare beneficial mutants. Similar outcomes were reported by Shahbazi et al. [18], who observed enhanced enzyme activity in certain strains only under low intensity or prolonged mutagenesis. Furthermore, Upton et al. [34] provided in silico evidence that beneficial phenotypes may emerge stochastically following transient declines in performance, supporting the nonlinear adaptive response observed in this study. A particularly noteworthy finding was the performance of the strain under UV for 4 min, which exhibited significantly enhanced cellulase activity after a relatively short exposure duration. These results align with growing evidence that brief UV treatments can effectively enhance enzyme activity. For example, Jafari et al. [35] optimized UV treatment at 3.7 min in *Aspergillus niger*, achieving a twofold increase in enzyme activity.

The strain obtained under optimal UV mutagenesis conditions (4 cm distance for 4 min exposure) was designated Mut-4 and subsequently cultivated in a 10 L stirred-tank bioreactor to assess its production performance. Mut-4 demonstrated significantly higher enzyme activities and protein concentrations than the original strain, with increases of up to 1.7-, 1.15-, and 1.91-fold for EG, BGL, and CBH, respectively. These findings indicate that the enhanced performance of Mut-4 was retained under pilot-scaled conditions, supporting its potential application in industrial bioprocesses.

In this study, the Mut-4 strain was cultivated using microcrystalline cellulose (Avicel) as the primary carbon source. Avicel is a highly crystalline, poorly soluble substrate known for its resistance to enzymatic hydrolysis and limited microbial accessibility [21]. Consequently, fermentation processes that utilize insoluble cellulose typically require extended cultivation times and vigorous agitation, often resulting in low biomass growth rates—factors that contribute to higher production costs [8]. To address these limitations, many high-yield cellulase production systems have incorporated soluble carbon sources, such as lactose (as inducers), or have employed mixed substrates to facilitate fermentation [13]. Despite these challenges, Mut-4 maintained robust growth and cellulase production without the use of soluble inducers or nutrient supplementation. This suggests an enhanced capacity for substrate utilization, presenting potential advantages for cost-effective and simplified bioprocessing [8].

Several high-performance cellulase-producing strains have been developed through genetic engineering, fed-batch fermentation strategies, and the use of soluble inducer sugars. For instance, *Trichoderma reesei* SEU-7 was derived from the RUT-C30 strain through *Agrobacterium*-mediated insertional mutagenesis to overexpress the endogenous β-glucosidase gene. When cultivated on lactose, this strain achieved a filter paper activity (FPase) of 47.0 U·mL^−1^ and BGL of 144.0 U·mL^−1^ [36]. However, this process required a fed-batch strategy involving mid-fermentation lactose supplementation (e.g., 3% pulse), along with precise pH and nutrient control to sustain enzyme induction. Another example is the RC-23-1 strain, developed through sequential chemical (diethyl sulfate) and UV mutagenesis of the RUT-C30 background. Under fed-batch cultivation with lactose as the carbon source, RC-23-1 exhibited high cellulase productivity, reaching enzyme activities of 182 FPU per gram of lactose consumed [37]. The enzyme cocktail produced by this strain was highly effective in converting approximately 60% of the glucan in pretreated rice straw into fermentable sugars. Nevertheless, this performance was strongly dependent on tightly controlled feeding strategies and optimized fermentation conditions. In contrast, the Mut-4 strain developed in this study was obtained solely through 4 min of UV exposure without any genetic modification. It achieved comparable enzyme activity in a simple batch process using crystalline cellulose as the sole carbon source without the need for soluble inducers or nutrient feeding. These findings underscore the robustness and practical advantages of Mut-4, particularly in terms of substrate utilization, process simplicity, and scalability for industrial applications.

## 5. Conclusions

This study showed that combining UV-induced mutagenesis with biochar supplementation is an effective strategy for enhancing cellulase production in *Trichoderma* sp. KMF006. Optimal UV conditions (4 cm distance and 4 min exposure), combined with the addition of biochar, led to the development of the Mut-4 strain. This mutant exhibited significantly improved enzyme activity and protein yield. In flask cultures, Mut-4 achieved 1.3- to 1.8-fold higher values compared with the original strain. These enhancements were sustained in a 10-L stirred-tank bioreactor, confirming the scalability and process stability of the mutant. Notably, maximum productivity was achieved within 2 weeks, resulting in a significant reduction in cultivation time.

This study also has some limitations. Although ITS sequencing was performed to confirm the identity of the Mut-4 strain, the specific mutations or regulatory mechanisms responsible for the enhanced phenotype remained uncharacterized, as no genome-wide analysis was conducted. Additionally, cellulase activity was evaluated only using Avicel, which does not fully capture the complexity of actual lignocellulosic biomass [21]. Therefore, further research should investigate the genetic basis of improved enzyme production using genome or transcriptome analysis. The performance of Mut-4 should also be evaluated using real biomass feedstocks, such as corn stover, bagasse, or wood [3]. Expanding this strategy to other enzyme systems may further clarify its broader applicability.

Nevertheless, this study presents a simple, low-cost, and nongenetically modified approach for enhancing fungal cellulase production. The consistent performance of Mut-4 under minimal process conditions underscores its potential as an industrial biocatalyst for sustainable lignocellulosic biomass conversion [8].

## 6. Patents

This study is associated with the patent application titled “Method for Producing Highly Active Cellulase, and Use of Highly Active Cellulase Produced Thereby”, which was filed by Kookmin University Industry-Academic Cooperation Foundation (Application No P-2024-181, filed on 19 November 2024) with the Korean Intellectual Property Office.

## Figures and Tables

**Table 1:** Maximum enzyme activities and protein concentrations of the UV-mutated strains under different exposure durations.

	EG Activity			BGL Activity			CBH Activity			Protein Concentration		
	U/mL	Std	Day ^(1)^	U/mL	Std	Day	U/mL	Std	Day	mg/mL	Std	Day
Original ^(2)^	34.750 ^c^	0.558	18	1.970 ^c^	0.249	21	0.593 ^b^	0.025	18	0.942 ^b^	0.021	21
UV 2 min ^(3)^	49.789 ^b^	1.716	15	3.309 ^a^	0.424	21	0.856 ^a^	0.043	21	1.234 ^a^	0.063	15
UV 4 min	64.599 ^a^	3.225	18	3.217 ^a,b^	0.911	21	0.823 ^a^	0.054	18	1.199 ^a^	0.057	21
UV 6 min	37.143 ^c^	7.222	18	2.462 ^b,c^	0.169	21	0.623 ^b^	0.125	18	0.892 ^b^	0.072	21
UV 8 min	63.251 ^a^	1.372	21	3.165 ^a,b^	0.009	21	0.804 ^a^	0.040	15	0.966 ^b^	0.010	21

^(1)^ The cultivation day on which the maximum value was observed is indicated for each variable. ^(2)^ Original refers to the unmutated *Trichoderma* strain. ^(3)^ Mutant strains were obtained through UV exposure for durations of 2–8 min. Superscript letters (a–c) denote significant differences between treatments within each column (*p* < 0.05).

## Data Availability

The data presented in this study are available from the corresponding author upon reasonable request.

## Abbreviations

The following abbreviations are used in this manuscript:

UV	Ultraviolet
EG	Endoglucanase
BGL	β-Glucosidase
CBH	Cellobiohydrolase
MEA	Malt Extract Agar
PDB	Potato Dextrose Broth
CMC	Carboxymethyl Cellulose
pNPG	*p*-Nitrophenyl-β-D-glucopyranoside
pNPC	*p*-Nitrophenyl-β-D-cellobioside
BSA	Bovine Serum Albumin
ITS	Internal Transcribed Spacer
NCBI	National Center for Biotechnology Information
SSF	Solid-State Fermentation
ANOVA	Analysis of Variance
FPase	Filter Paper Activity

## Appendix A. Maximum Cellulase Activities and Protein Concentrations of Trichoderma Strains

**Table A1:** Maximum cellulase activities and protein concentrations of *Trichoderma* strains subjected to varying UV light distances.

	EG Activity			BGL Activity			CBH Activity			Protein Concentration		
	U/mL	Std	Day ^(1)^	U/mL	Std	Day	U/mL	Std	Day	mg/mL	Std	Day
Original ^(2)^	21.824 ^b^	1.406	21	1.926 ^c^	0.102	21	0.538 ^b^	0.017	21	0.341 ^c^	0.042	15
UV 4 cm ^(3)^	33.784 ^a^	4.757	18	2.432 ^a,b^	0.051	15	0.458 ^b^	0.012	18	0.936 ^a^	0.012	18
UV 9 cm	28.593 ^a^	2.197	21	2.066 ^b,c^	0.130	21	0.651 ^a^	0.060	21	0.880 ^a^	0.008	21
UV 13 cm	29.451 ^a^	1.874	21	2.626 ^a^	0.305	21	0.515 ^b^	0.072	21	0.803 ^b^	0.036	18

^(1)^ The cultivation day on which the maximum value was observed for each variable. ^(2)^ Original refers to the unmutated *Trichoderma* strain. ^(3)^ Mutant strains were induced by varying the distance from the UV light source (4, 9, and 13 cm). Superscript letters (a–c) indicate significant differences between treatments within each column (*p* < 0.05).

## References

[1] Ejaz U., Sohail M., Ghanemi A. (2021). Cellulases: From bioactivity to a variety of industrial applications. Biomimetics.

[2] Kuhad R.C., Gupta R., Singh A. (2011). Microbial cellulases and their industrial applications. Enzym. Res..

[3] Asgher M., Ahmad Z., Iqbal H.M.N. (2013). Alkali and enzymatic delignification of sugarcane bagasse to expose cellulose polymers for saccharification and bio-ethanol production. Ind. Crops Prod..

[4] Iqbal H.M.N., Ahmed I., Zia M.A., Irfan M. (2011). Purification and characterization of the kinetic parameters of cellulase produced from wheat straw by *Trichoderma viride* under SSF and its detergent compatibility. Adv. Biosci. Biotechnol..

[5] Iqbal H.M.N., Kyazze G., Keshavarz T. (2013). Advances in the valorization of lignocellulosic materials by biotechnology: An overview. BioResources.

[6] Elakkiya P., Muralikrishnan V. (2014). Cellulase production and purification of mutant strain *Trichoderma viride*. Int. J. Curr. Microbiol. Appl. Sci..

[7] Kant S., Das S., Roy S., Tripathy S. (2024). Fungal cellulases: A comprehensive review. Nucleus.

[8] Siqueira J.G.W., Rodrigues C., de Souza Vandenberghe L.P., Woiciechowski A.L., Soccol C.R. (2020). Current advances in on-site cellulase production and application on lignocellulosic biomass conversion to biofuels: A review. Biomass Bioenergy.

[9] Xu Q., Singh A., Himmel M.E. (2009). Perspectives and new directions for the production of bioethanol using consolidated bioprocessing of lignocellulose. Curr. Opin. Biotechnol..

[10] Pribowo A.Y., Hu J., Arantes V., Saddler J.N. (2013). The development and use of an ELISA-based method to follow the distribution of cellulase monocomponents during the hydrolysis of pretreated corn stover. Biotechnol. Biofuels.

[11] Lynd L.R., Weimer P.J., van Zyl W.H., Pretorius I.S. (2002). Microbial cellulose utilization: Fundamentals and biotechnology. Microbiol. Mol. Biol. Rev..

[12] Horn S.J., Vaaje-Kolstad G., Westereng B., Eijsink V. (2012). Novel enzymes for the degradation of cellulose. Biotechnol. Biofuels.

[13] Ellilä S., Fonseca L., Uchima C., Cota J., Goldman G.H., Saloheimo M., Sacon V., Siika-aho M. (2017). Development of a low-cost cellulase production process using Trichoderma reesei for Brazilian biorefineries. Biotechnol. Biofuels.

[14] Klein-Marcuschamer D., Oleskowicz-Popiel P., Simmons B.A., Blanch H.W. (2012). The challenge of enzyme cost in the production of lignocellulosic biofuels. Biotechnol. Bioeng..

[15] Liu P., Lin A., Zhang G., Zhang J., Chen Y., Shen T., Zhao J., Wei D., Wang W. (2019). Enhancement of cellulase production in *Trichoderma reesei* RUT-C30 by comparative genomic screening. Microb. Cell Fact..

[16] Parekh S., Vinci V.A., Strobel R.J. (2000). Improvement of microbial strains and fermentation processes. Appl. Microbiol. Biot..

[17] Fang K., Ma J., Wang X., Xu Z., Zhang Z., Li P., Wang R., Wang J., Sun C., Dong Z. (2023). Flow-cytometric cell sorting coupled with UV mutagenesis for improving pectin lyase expression. Front. Bioeng. Biotechnol..

[18] Shahbazi S., Shams G., Tabandeh F., Nahvi I. (2014). Gamma and UV radiation induced mutagenesis in *Trichoderma reesei* to enhance cellulases enzyme activity. Int. J. Farming Allied Sci..

[19] Awad Y.M., Lee S.S., Kim K.-H., Ok Y.S., Kuzyakov Y. (2018). Carbon and nitrogen mineralization and enzyme activities in soil aggregate-size classes: Effects of biochar, oyster shells, and polymers. Chemosphere.

[20] Wang J., Wang S. (2019). Preparation, modification and environmental application of biochar: A review. J. Clean. Prod..

[21] Zhang Y., Wang J., Feng Y. (2021). The effects of biochar addition on soil physicochemical properties: A review. CATENA.

[22] Myeong S., Yun J. (2024). Culture of Trichoderma Sp. with biochar to produce high-activity cellulase in a laboratory. BioResources.

[23] Niu S., Li C., Gao S., Tian J., Zhang C., Li L., Huang Y., Lyu H. (2024). Biochar, microbes, and biochar-microbe synergistic treatment of chlorinated hydrocarbons in groundwater: A review. Front. Microbiol..

[24] Kim Y., Park S., Kim Y. (2018). Trichoderma sp., KMF006 Strain Producing Cellulase with High Activity. Korea Patent.

[25] Nelson N. (1944). A photometric adaptation of the Somogyi method for the determination of glucose. J. Biol. Chem..

[26] Joo A.-R., Jeya M., Lee K.-M., Sim W.-I., Kim J.-S., Kim I.-W., Kim Y.-S., Oh D.-K., Gunasekaran P., Lee J.-K. (2009). Purification and characterization of a β-1,4-glucosidase from a newly isolated strain of *Fomitopsis pinicola*. Appl. Microbiol. Biot..

[27] Kredics L., Antal Z., Doczi I., Manczinger L., Kevei F., Nagy E. (2003). Clinical importance of the genus Trichoderma. Acta Microbiol. Immunol. Hung..

[28] Pérez J., Muñoz-Dorado J., de la Rubia T., Martínez J. (2002). Biodegradation and biological treatments of cellulose, hemicellulose and lignin: An overview. Int. Microbiol..

[29] Rana V. (2014). Optimization of Enzymatic Hydrolysis of Lignocellulosic Biomass. Ph.D. Thesis.

[30] Zhang X., Yao Y., Madzak C., Du G., Zhou J., Chen J. (2020). TlSWO, a novel swollenin from *Talaromyces leycettanus* JCM12802, enhances enzymatic hydrolysis of lignocellulose. Biotechnol. Biofuels.

[31] Santos C.A., Morais M.A., Terrett O.M., Lyczakowski J.J., Zanphorlin L.M., Ferreira-Filho J.A., Tonoli C.C., Murakami M.T., Dupree P., Souza A.P. (2017). An engineered GH5 endoglucanase from *Trichoderma harzianum* and its synergistic action with a swollenin. Biotechnol. Biofuels.

[32] Yao G., Wu R., Kan Q., Gao L., Yang J., Huang J., Zhou J., Chen J., Du G. (2016). A strategy for enhancing the production of cellulolytic enzymes in *Trichoderma reesei* by overexpression of the global transcription factor ACE3. J. Ind. Microbiol. Biotechnol..

[33] Qin Y., Wei X., Song X., Wang L., Zhang L., Li X., Peng Y., Jiang Z., Li H. (2020). Engineering a cellulase cocktail based on Swollenin addition to enhance lignocellulose saccharification. Bioresour. Technol..

[34] Upton D.J., McQueen-Mason S.J., Wood A.J. (2020). In silico evolution of *Aspergillus niger* organic acid production suggests strategies for switching acid output. Biotechnol. Biofuels.

[35] Jafari N., Jafarizadeh-Malmiri H., Hamzeh-Mivehroud M., Adibpour M. (2017). Optimization of UV irradiation mutation conditions for cellulase production by mutant fungal strains of *Aspergillus niger* through solid state fermentation. Green Process. Synth..

[36] Li C., Zhao J., Yang Y., Rojas O.J. (2017). Development of *Trichoderma reesei* mutant SEU-7 for enhanced cellulase production using lactose in fed-batch fermentation. Biotechnol. Biofuels.

[37] Adsul M.G., Dixit P., Saini J.K., Gupta R.P., Ramakumar S.S.V., Mathur A.S. (2022). Morphologically favorable mutant of *Trichoderma reesei* for low viscosity cellulase production. Biotechnol. Bioeng..

